# A Case Report of Hemophagocytic Lymphohistiocytosis (HLH) - An Unusual Complication of Dengue Infection

**DOI:** 10.7759/cureus.26504

**Published:** 2022-07-01

**Authors:** Sourya Acharya, Samarth Shukla, Tushar Sontakke, Irhsad VS, Charan Bagga, Sameera Dronamraju, Anamika Giri

**Affiliations:** 1 Department of Medicine, Datta Meghe Institute of Medical Sciences, Wardha, IND; 2 Department of Medicine, Jawaharlal Nehru Medical College, Wardha, IND; 3 Department of Pathology, Datta Meghe Institute of Medical Sciences, Wardha, IND; 4 Department of Pathology, Jawaharlal Nehru Medical College, Wardha, IND; 5 Department of Medicine, Datta Meghe Institute of Medical Science, Wardha, IND; 6 Department of Internal Medicine, Jawaharlal Nehru Medical College, Wardha, IND

**Keywords:** neoplasm, autosomal, autoimmune, hyperferritinemia, cytotoxic

## Abstract

Hemophagocytic lymphohistiocytosis (HLH) is an autoimmune phenomenon characterized by reactive hyperactivity of cytotoxic T cells and histiocytes, leading to hypercytokinemic injury to cells and organ system, which leads to multiorgan dysfunction and ultimate failure. Epstein-Barr virus (EBV) is most commonly associated with secondary HLH with high mortality, but increasing evidence suggests the association of the dengue virus. When associated with dengue infection, it carries a grave prognosis and correlates with the disease severity. Furthermore, it overlaps with dengue sepsis, so it can often be misdiagnosed as sepsis. Typically the patients have hyperferritinemia, hypertriglyceridemia, transaminitis, and marrow features suggestive of hemophagocytosis. The treatment is usually systemic corticosteroids, intravenous immunoglobulin, and chemotherapy with etoposide. We present a case of a 25-year-old male patient who had a dengue infection and further developed HLH with pulmonary infiltrates. Clinical suspicion alerted us to look for other evidence of HLH on the fourth day of admission, and appropriate investigations were done. Diagnosis of HLH was confirmed by HLH-2004, HScore criteria, and bone marrow aspirate examination. Treatment was given in the form of corticosteroids and chemotherapy along with other supportive measures. The patient responded to the line of management.

## Introduction

Hemophagocytic lymphohistiocytosis (HLH) is a hyper immunoinflammatory, hyperferritinemic condition characterized by uncontrolled activation and proliferation of cytotoxic T lymphocytes and histiocytes that secrete large amounts of inflammatory cytokines.

Hemophagocytosis is done by activated macrophages. The hypercytokinemia leads to multiorgan dysfunction and failure. The aetiology of HLH may be classified as genetic (primary) and acquired (secondary). Familial HLH usually follows an autosomal recessive inheritance. About 40 to 60 percent of the mutations occur in PRF 1 and Unc-13 Homolog D (UNC13D) genes. Other genes involved are Syntaxin 11 (STX 11) and Syntaxin Binding Protein 2 (STXBP2) [1,2]. Secondary HLH is usually acquired by viral infections, immunodeficiency states, autoimmune disorders, and cancers.

Epstein-Barr virus (EBV) is the most common agent to cause HLH, which has poor outcomes [3]. Currently, there is increasing data that implicate that severe dengue virus infection also causes secondary HLH with poor outcomes. The mortality may increase up to 43% [4,5].

## Case presentation

A 25-year-old male presented to us with a history of fever, headache, myalgia, and vomiting for a duration of two days. There was no history of diplopia, blurring of vision, seizures, hematemesis, melena, and cough. He did not have a COVID-19 infection, and he had been administered the first dose of the COVISHIELD™ (AstraZeneca, Cambridge, United Kingdom) vaccine one month back.

On examination, pulse was 112/minute, regular, blood pressure (BP) was 92/60 mmHg, respiratory rate was 24 breaths/minute, and Oxygen saturation was 98% while breathing ambient air. There were no icterus, cyanosis, edema, or rash on skin. Per-abdomen examination revealed grade 1 soft non-tender splenomegaly and soft non-tender hepatomegaly 2 cm below the right costal margin. Cardiovascular, respiratory, and central nervous system examination was normal. He was admitted and resuscitated with intravenous crystalloid fluid of 50mL/kg, which resulted in the successful resolution of hypotension.

**Figure 1:**
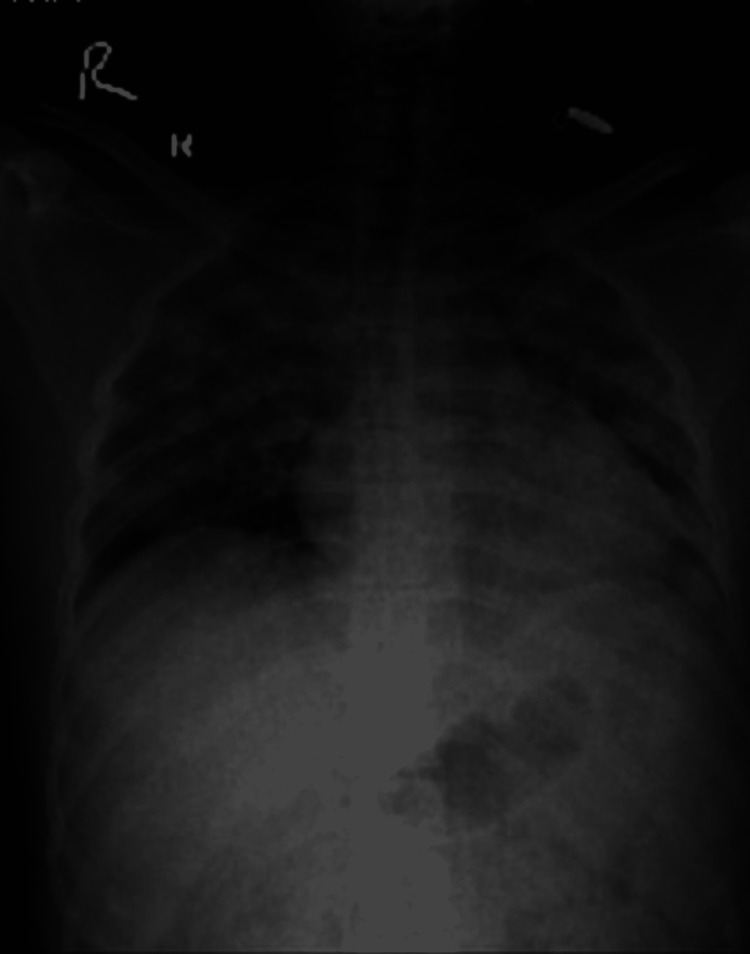
Chest X-ray showing bilateral infiltrates in lung fields

**Figure 2:**
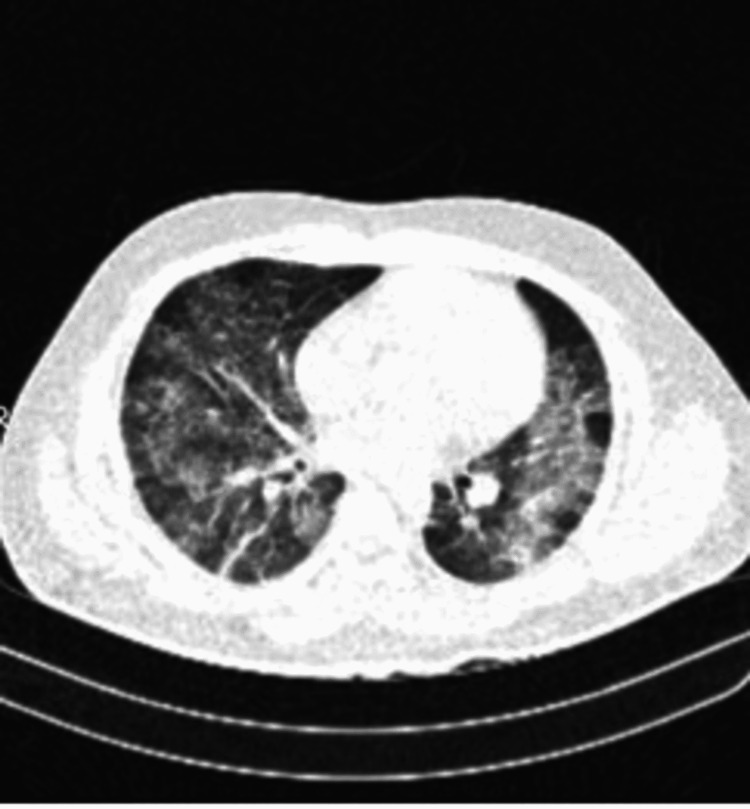
HRCT of lungs showing multiple ill-defined patchy ground-glass opacities with septal thickening and patchy areas of consolidation in bilateral lung fields HRCT - high-definition computed tomography

Investigations revealed hemoglobin (Hb) 9 gram %, total leucocyte count (TLC) 2000/mm 3, and differential leukocyte count: neutrophils 58%, lymphocytes 40%, monocyte 1%, basophil 1%. The absolute platelet count was 84*10 9/L. Peripheral smear was negative for malaria parasites. Paracheck® (Orchid Biomedical Systems, Goa, India) for plasmodium falciparum was negative, IgM scrub typhus was negative, and dengue nonstructural protein 1 (NS1) was positive. The kidney function test was normal. Liver function test showed serum bilirubin 1.8 mg%, alanine transaminase (ALT) 220 U/L, and aspartate aminotransferase (AST) 140 U/L. Empirical antibiotics were initiated for fever. Blood culture and urine culture did not show any bacterial growth. On the second day, the fever was persistent, and the patient developed tachypnoea. SpO2 was 88% in room air and PaO2/FiO2 was 300. SARS-CoV 2 reverse transcription polymerase chain reaction (RT-PCR) was negative. Supplemental oxygen therapy was initiated with 2 liters of oxygen through a nasal cannula to maintain oxygen saturation of 95%. In the next 12 hours, oxygen requirement increased to 8 liters to maintain saturation over 95%; at this moment, non-invasive ventilation was initiated. A chest X-ray (CXR) revealed bilateral infiltrates (Figure 1), and a high-resolution computed tomography (HRCT) scan showed bilateral ground-glass opacities (Figure 2). Two-dimensional echo and serum D-dimer were normal.

Antibiotics were upgraded to IV piperacillin plus tazobactam and levofloxacin. Supportive treatment was continued. From the second until the seventh day in the hospital, the fever continued. Serial blood investigations were done (Table 1)

**Table 1:** Trend of laboratory investigations of the patient ALT - alanine transaminase, AST - aspartate aminotransferase, LDH - lactate dehydrogenase

	Day 1	Day 2	Day 3	Day 4	Day 5	Day 6	Day 7	Day 8	Day 9	Day 10
Hemoglobin (gm/dl)	8.8	8.3	8.2	7.2	7.5	7.5	7.8	8	8.2	8.6
Hematocrit (36-46)	38	39	44	46	44	44	43	41	40	37
White cell count (per microlitre)	4000	3500	2600	1600	1700	1800	2000	3100	4200	6300
Platelet count (10^9 ^per microlitre)	55	45	41	33	36	36	42	49	60	110
ALT (U/L)	142	156	189	201	200	190	179	109	84	61
AST (U/L)	112	120	124	146	144	140	108	89	74	46
Creatinine (mg/dl)	1.5	1.7	1.8	2.4	2.2	1.9	1.5	1.2	0.9	0.8
LDH (U/L)	-	-	750	1107	1110	850	240	224	176	171
Ferritin (ng/ml)	-	-	1121	1300	1000	997	956	700	386	211
Triglyceride (mg/dl)	-	-	291	286	284	270	270	266	240	231
Fibrinogen (g/dl)	-	-	87	92	84	75	74	63	61	59

**Figure 3:**
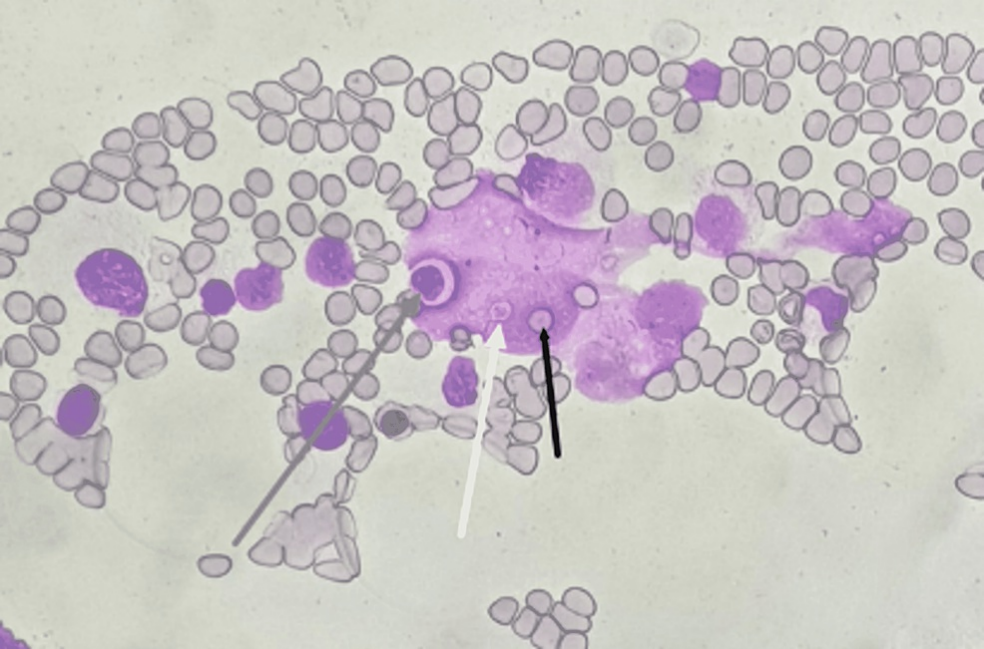
High power fields (400x) of a bone marrow aspirate smear demonstrating histiocytes showing features of hemophagocytosis of the leucocytes (grey arrow), platelets (white arrow), and mature erythrocytes (black arrow), features suggestive of HLH HLH - hemophagocytic lymphohistiocytosis

**Figure 4:**
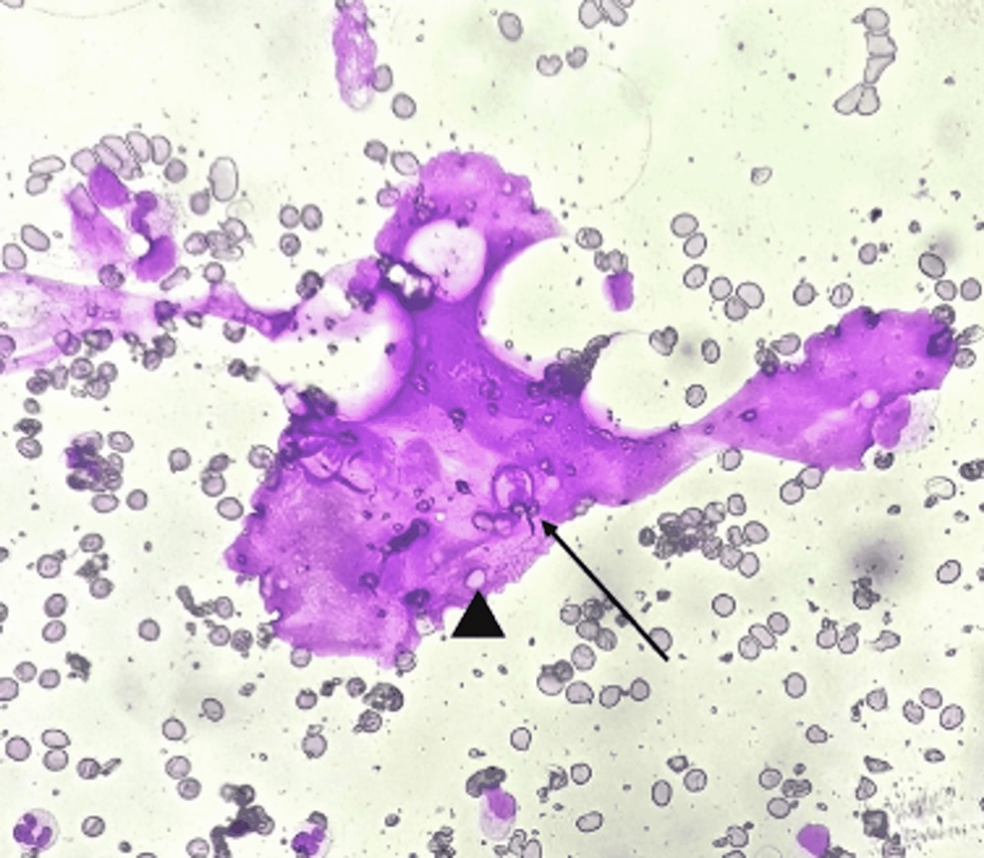
High power fields (400x) of a bone marrow aspirate smear demonstrating a histiocyte with an engulfed neutrophil (arrow) and mature erythrocytes within (arrowhead), suggesting hemophagocytic lymphohistiocytosis.

On day four, serum ferritin level and triglyceride level were done, which showed marked elevation. A clinical possibility of HLH was made, and an HScore was calculated to be 183, with an HLH probability of 80%. A bone marrow aspiration was done, which suggested features of HLH. (Figures 3, 4)

IV dexamethasone was started with a dose of 10mg/m2/d, and IV etoposide was initiated at a dose of mg intravenous over one hour twice a week for two weeks and once weekly for six weeks. The patient showed clinical recovery by day eight; laboratory parameters improved from day seven, and he became afebrile from day eight. The patient was discharged after the third dose of etoposide. He was advised to continue visits for the remaining six weeks for completion of the total etoposide course. In the follow-up, after one month of the last dose of etoposide, all the hematologic parameters were normal, and the patient was doing well.

## Discussion

Dengue virus belongs to the genus flavivirus within the Flaviviridae family. There are four distinct serotypes. The virus causes a dengue infection. The complications of dengue fever (DF) range from thrombocytopenia, and sepsis, to shock syndrome.

HLH is a rare complication of dengue infection and can be often missed if not suspected because it may mimic sepsis syndrome. As per HLH-2004 diagnostic criteria, HLH is diagnosed when at least five of the eight criteria listed are fulfilled. These criteria are fever, splenomegaly, cytopenia affecting at least two of three lineages in peripheral blood, ferritin ≥500 μg/L, hypertriglyceridemia and/or hypofibrinogenemia, hemophagocytosis in bone marrow or spleen or lymph nodes, low or absent natural killer (NK) cell activity, and high level of soluble interleukin-2 receptor alpha chain (CD25) [6]

Hyperferritinemia is strongly associated with HLH, and a cutoff value of >10,000mcg/L is 90% sensitive and 96% specific for its diagnosis. It also correlates with disease activity. This emphasizes the need for closer monitoring in dengue virus-infected patients with hyperferritinemia [7] HScore is another criterion used for the diagnosis of HLH. A cutoff of 168 points reveals a sensitivity of 100% and a specificity of 94.1%. Our patient had a HScore of 183 [8]. Along with other complications of dengue fever, superadded HLH may lead to increase mortality. Patients with severe dengue are also at risk of developing secondary HLH, which would further contribute to the high mortality [9]. HLH is a hyperinflammatory state that induces cytokine storms, leading to multiorgan dysfunction. The bone marrow in HLH shows macrophage-derived phagocytosis of blood cells [10]

Lung involvement in HLH is not frequently described. In a study of 219 patients with HLH, 54% had lung involvement. The patients with lung involvement had serious multiorgan dysfunction [11]. The various radiologic findings included were centrilobular nodules, consolidations, ground-glass opacities, septal thickening, pleural effusions, and mediastinal lymphadenopathy. Our patient had six out of eight criteria for HLH with pulmonary involvement in the form of ground-glass opacities, consolidations, and septal thickening. A bone marrow aspiration was done which showed hemophagocytosis of platelets, erythrocytes, and leukocytes, features suggestive of HLH.

The treatment approach of HLH is targeted first toward the inciting event, which may be a viral infection, autoimmune disorder, or malignancy. The HLH-94 protocol states that induction therapy with etoposide is indicated in malignancy-associated HLH or where there is evidence of clinical deterioration. Other therapies are corticosteroids, intravenous immunoglobulin, and cyclosporin. Allogenic stem cell transplantation is reserved for refractory cases [12,13].

## Conclusions

Hemophagocytic lymphohistiocytosis is an immunologically mediated inflammatory response to viral infections, immune disorders, and malignancy. HLH induces cytokine storms, and the bone marrow shows lymphohistiocytic reaction and macrophagic hemophagocytosis. Though EBV is the most common viral infection known to cause HLH, dengue-indued HLH is being largely reported. Association of HLH with dengue fever is an indicator of severe disease. Pulmonary involvement though rare but can occur in HLH. It may be missed in dengue infection because of the simultaneous presence of septicemia in dengue, so a high index of suspicion is required. HLH is diagnosed using the HLH-2004 and HScore criteria. Treatment focuses on targeting the primary pathology. Drugs used in the treatment of HLH are corticosteroids, intravenous immunoglobulin, and etoposide.
